# A Rare Cause of Low Back Pain

**DOI:** 10.7759/cureus.42647

**Published:** 2023-07-29

**Authors:** Abigail Conroy, Justin Bui, Emily Barnard, Lazaro Diaz

**Affiliations:** 1 Dr. Kiran C. Patel College of Allopathic Medicine, Nova Southeastern University, Davie, USA; 2 Graduate Medical Education (GME) Consortium, Hospital Corporation of America (HCA) Florida Kendall Hospital, Miami, USA; 3 Department of Internal Medicine, Hospital Corporation of America (HCA) Florida Kendall Hospital, Miami, USA

**Keywords:** mri, idiopathic lumbar disorder, back pain, idiopathic spinal epidural lipomatosis, lumbar epidural lipomatosis, spinal epidural lipomatosis

## Abstract

This is the case of a 60-year-old man with no known medical history who presented with progressively worsening lumbar pain and was found to have idiopathic dorsal epidural lipomatosis. The patient’s condition improved significantly with pain management. Therefore, no surgical intervention was warranted at the time, but the patient was advised to keep close follow-up as an outpatient. Being familiar with this potential cause of lumbar pain is vital, as it can lead to severe morbidity if left unrecognized.

## Introduction

Spinal epidural lipomatosis (SEL) is a rare condition characterized by the accumulation of unencapsulated fat in the extradural space, causing compression of the spinal cord and surrounding neural structures. The exact pathology of SEL is not yet fully understood, but it is commonly observed in patients exposed to exogenous glucocorticoids [1]. The most common cause of SEL not associated with exogenous steroid administration is obesity [1]. SEL has also been seen in patients with Cushing's syndrome and metabolic syndrome [1].

The first case of SEL was described in 1975 by Lee et al. in a patient on a long-term exogenous steroid regimen [2]. Studies have since found the annual incidence of SEL to be about 2.5%, with a higher prevalence in males and overweight patients [3]. The diagnosis of SEL is made by MRI, which can visualize the overgrowth of epidural adipose tissue. We aim to highlight this rare cause of back pain, as recognition and proper management of this disease can prevent extensive disease progression.

## Case presentation

We present the case of a 60-year-old man with no known past medical history who presented with progressively worsening lumbar pain, fever, and chills over the past 48 hours. The pain began after the patient lifted a heavy item and was exacerbated with further movement. The patient found no relief from cyclobenzaprine, aspirin, or a steroid injection he received from urgent care the previous night. He denied saddle anesthesia, lower extremity numbness, or tingling. He denied any other symptoms, including headaches, neck stiffness, nausea, vomiting, abdominal pain, diarrhea, or urinary symptoms. The physical examination was benign and only notable for lumbar paraspinal tenderness and a positive bilateral straight leg test. No vertebral tenderness was noted.

In the ED, the patient was noted to be febrile to 38.7 °C, with a blood pressure of 124/75, a heart rate of 108 beats per minute, a respiratory rate of 38, an O2 saturation of 98% on room air, and a BMI of 38. Blood cultures were ordered. Lactic acid was initially 2.9 but came down to 0.9 after IV fluid resuscitation. The patient's white blood cell count was elevated at 20 × 109/L. All other labs, including basic metabolic panel (BMP), thyroid-stimulating hormone (TSH), creatinine, liver function tests, and serum lipid profile, were within normal limits, with the exception of a mildly elevated non-fasting glucose on admission at 132 mg/dL and a triglyceride of 143 mg/dL. This patient was admitted to the hospital for sepsis workup and treatment.

**Figure 1:**
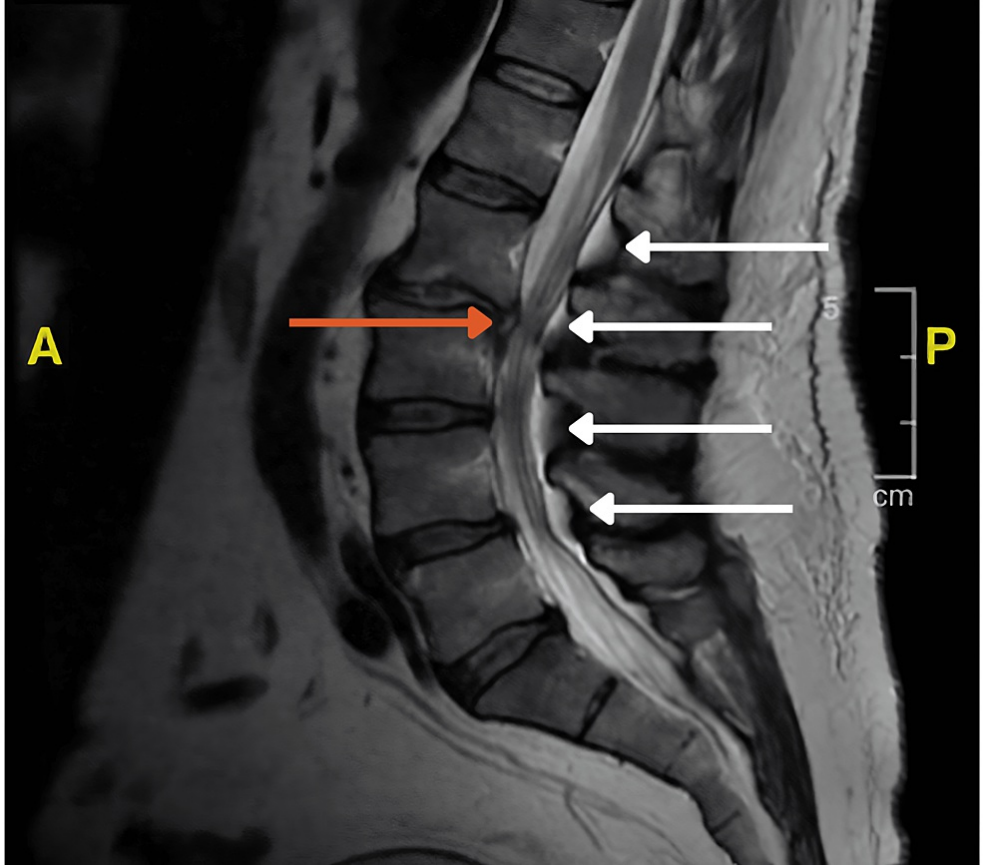
MRI T2 with contrast of the lumbosacral spinal cord showing fat compression at multiple levels (white arrows). L2-L3 disc herniation (orange arrow).

Lumbar MRI showed thecal sac compression at multiple levels and dorsal epidural lipomatosis aggravated by degenerative changes and an L2-L3 disc herniation (Figure 1).

Blood cultures came back positive for Streptococcus intermedius in one of two sets sensitive to vancomycin and ceftriaxone. An investigation into the source was unrevealing. Additional labs, including urine-free cortisol and late-night salivary cortisol, were within normal limits. The patient was started on Ceftriaxone 2 g IV q24hr and completed a 14-day course.

The patient’s condition improved significantly with antibiotic use and non-opioid pain management. Therefore, no surgical intervention was warranted at the time. The patient was advised to keep close follow-up as an outpatient, and lifestyle modifications, including weight reduction, were recommended.

## Discussion

SEL can cause non-specific symptoms, including myelopathy, radiculopathy, sensory disturbances, and claudication [4]. While symptoms may appear suddenly, as in this case, they typically develop over several months to years [3]. Unfortunately, these symptoms are often mistaken for other causes of lower back pain, such as muscle strain and disc herniation.

Treatment for SEL consists of addressing the underlying pathology, decreasing exogenous steroid use, and lifestyle modification. If symptoms are persistent or severe, surgical decompression of the accumulated adipose tissue is warranted [5]. Pain relief can be achieved with medications like nonsteroidal anti-inflammatory drugs (NSAIDs) or opioids. Still, surgery is often the preferred option for long-term improvement [6,7]. Fortunately, most patients who undergo surgery experience significant relief from their symptoms.

Corticosteroid injections into the spinal epidural space for lower back pain can result in the development or worsening of SEL [8]. Although most SEL cases are caused by oral steroid use, local steroid injections have also been linked to several reported cases [9].

Significant complications can arise from SEL when the burden of fat accumulation becomes substantial enough to compress the spinal cord. The complications of SEL can manifest as neurological dysfunction such as radiculopathy, neurogenic claudication, cauda equina syndrome, or even progressive paraplegia [8].

## Conclusions

This case of idiopathic SEL highlights the importance of recognizing this rare cause of lower back pain. SEL can be unresponsive to standard lumbar pain therapy and may even be aggravated by some treatments, like steroid injections. Being familiar with this potential cause of lumbar pain is vital, as it can lead to severe morbidity if left unrecognized. Close monitoring and follow-up are recommended, as surgical intervention may be required if symptoms persist or worsen.
